# THE ROLE OF RETIREMENT IN COGNITIVE AGING: RESULTS FROM THE SWEDISH ADOPTION/TWIN STUDY OF AGING

**DOI:** 10.1093/geroni/igz038.092

**Published:** 2019-11-08

**Authors:** Ross Andel, Deborah Finkel, Nancy L Pedersen

**Affiliations:** 1 University of South Florida, Tampa, Florida, United States; 2 Indiana University Southeast, New Albany, Indiana, United States; 3 Karolinska Institutet, Stockholm, Stockholms Lan, Sweden

## Abstract

Retirement is a major life transition that may influence the aging process. Using a two-slope growth curve model with retirement age as the pivot point, we studied change in major cognitive domains before and after retirement. Participants were 393 members of the Swedish Adoption/Twin Study of Aging who retired after the age of 50 (mean=63 years, range 51-75 years) and who were followed for over 20 years with seven testing occasions. After controlling for age, sex, and education, we observed no change in memory pre-retirement (p=.935) and significant memory decline post-retirement (Estimate=-0.17, p=.001); decline in speed, which more than doubled after retirement (Estimate=-0.20, p=.001 before, Estimate=0.54, p<.001 after); improvement in verbal abilities before (Estimate=0.15, p=.020) and decline in verbal abilities after retirement (Estimate=-0.11, p<.001); and some decline in spatial abilities before (Estimate=-0.18, p=.059) followed by accelerated decline after retirement (Estimate=-0.33, p<.001). Retirement may be a good target for intervention.

